# Seven novel mutations in the long isoform of the *USH2A* gene in Chinese families with nonsyndromic retinitis pigmentosa and Usher syndrome Type II

**Published:** 2011-06-09

**Authors:** Wenjun Xu, Hanjun Dai, Tingting Lu, Xiaohui Zhang, Bing Dong, Yang Li

**Affiliations:** Beijing Institute of Ophthalmology, Beijing Tongren Eye Center, Beijing Tongren Hospital, Capital Medical University, Beijing Ophthalmology & Visual Sciences Key Laboratory, Beijing, China

## Abstract

**Purpose:**

To describe the clinical and genetic findings in one Chinese family with autosomal recessive retinitis pigmentosa (arRP) and in three unrelated Chinese families with Usher syndrome type II (USH2).

**Methods:**

One family (FR1) with arRP and three unrelated families (F6, F7, and F8) with Usher syndrome (USH), including eight affected members and seven unaffected family individuals were examined clinically. The study included 100 normal Chinese individuals as normal controls. After obtaining informed consent, peripheral blood samples from all participants were collected and genomic DNA was extracted. Genotyping and haplotyping analyses were performed on the known genetic loci for arRP with a panel of polymorphic markers in family FR1. In all four families, the coding region (exons 2–72), including the intron-exon boundary of the *USH2A* (Usher syndrome type −2A protein) gene, was screened by PCR and direct DNA sequencing. Whenever substitutions were identified in a patient, a restriction fragment length polymorphism (RFLP) analysis, single strand conformation polymorphism (SSCP) analysis, or high resolution melt curve analysis (HRM) was performed on all available family members and on the 100 normal controls.

**Results:**

The affected individuals presented with typical fundus features of retinitis pigmentosa (RP), including narrowing of the vessels, bone-spicule pigmentation, and waxy optic discs. The electroretinogram (ERG) wave amplitudes of the available probands were undetectable. Audiometric tests in the affected individuals in family FR1 were normal, while indicating moderate to severe sensorineural hearing impairment in the affected individuals in families F6, F7, and F8. Vestibular function was normal in all patients from all four families. The disease-causing gene in family FR1 was mapped to the *USH2A* locus on chromosome 1q41. Seven novel mutations (two missenses, one 7-bp deletion, two small deletions, and two nonsenses) were detected in the four families after sequencing analysis of *USH2A*.

**Conclusions:**

The results further support that mutations of *USH2A* are also responsible for non-syndromic RP. The mutation spectrum among Chinese patients might differ from that among European Caucasians.

## Introduction

Retinitis pigmentosa (RP) is a heterogeneous group of retinal dystrophies, characterized by progressive degeneration of the photoreceptors. Clinical features include progressive night blindness, constriction and gradual loss of the peripheral visual field, and eventual loss of visual acuity. With an incidence of 1 in 3,500, RP can be inherited as an autosomal recessive (arRP), an autosomal dominant (adRP), or an X-linked recessive (xlRP) pattern [1,2].

RP can be classified as syndromic and nonsyndromic RP, based on whether or not extra-ocular diseases exist. Nonsyndromic arRP is caused by the mutations of 32 identified genes [1,2]. Syndromic RP includes more than 30 different syndromes [1,2]. The most common syndrome is Usher syndrome (USH), which is also an autosomal recessive disorder characterized by sensorineural hearing loss, variable vestibular dysfunction, and visual impairment due to retinitis pigmentosa [2,3]. Clinically, USH is subdivided into three types: USH type I (USH1), USH type II (USH2), and USH type III (USH3). USH1 is the most severe form of this disease and is characterized by congenital profound hearing loss, prepuberal onset of RP, and vestibular dysfunction. Patients with USH2 experience congenital moderate to severe hearing impairment, and postpuberal onset of RP with intact vestibular function. Patients with USH3 show progressive postlingual hearing loss, later onset of RP, and variable vestibular dysfunction. Of the three clinical types, USH2, which accounts for more than half of all patients with USH, is the most common form of USH [2-4]. To date, reports indicate that three genes (*USH2A* [Usher syndrome type −2A protein], *GPR98* [G-protein coupled receptor 98], and *DFNB31* [CASK-interacting protein CIP98 isoform 1]) are responsible for USH2, and most USH2 patients have mutations in *USH2A* [3-9].

*USH2A*, located on chromosome 1q41, has two alternatively spliced isoforms: a short *USH2A* isoform a, consisting of 21 exons, and a long *USH2A* isoform b, consisting of 51 additional exons at the 3′ end of *USH2A* [5,9]. The protein usherin, encoded by USH2A isoform b, is a transmembrane protein, which has 5,202 amino acids [9]. The usherin is transiently expressed in the stereocilia of cochlear hair cells, suggesting an important role in their maturation [4,9-11]. In mammalian photoreceptors, the usherin is expressed specifically in the connecting cilia, which links the inner and outer retinal segments; this would appear to indicate that it is crucial for the long-term maintenance of photoreceptors [9-11].

Since identification of *USH2A*, several studies have indicated that mutations of this gene can cause a significant proportion of non-syndromic recessive RP [12-20].

This study investigated a Chinese family with non-syndromic arRP. After haplotyping analysis, the disease-causing gene was mapped to the *USH2A* region. Mutations screening of the *USH2A* gene, corresponding to the *USH2A* isoform b, was then performed in this nonsyndromic RP family and in three USH2 families. Seven novel mutations were identified.

## Methods

### Clinical data and sample collection

This study adhered to the tenets of the Declaration of Helsinki for research involving human subjects. The Beijing Tongren Hospital Joint Committee on Clinical Investigation approved the study. One Chinese family with nonsyndromic RP and three unrelated Chinese families with USH were referred to Beijing Tongren Hospital. After informed consent was obtained, each participant underwent careful ophthalmologic examinations, including best-corrected visual acuity testing using E decimal charts, slit-lamp biomicroscopy, fundus examination with dilated pupils, visual field testing, and electroretinogram (ERG) examination. Three probands from the three families with USH underwent audiometric testing, including otoscopy and standard pure-tone audiometry, and vestibular tests. The patients with nonsyndromic arRP were given audiometric tests after the disease gene was mapped to chromosome 1q41, where the *USH2A* gene is located. Clinical diagnosis of USH2 was based on the clinical history, typical RP fundus appearance, sensorineural hearing impairment, and intact vestibular function. Peripheral blood was obtained by venipuncture, and genomic DNA was extracted according to standard phenol protocols.

### Genotyping and haplotyping analysis

Genotyping was performed with 50 microsatellite markers from autosomes for the known arRP loci in family FR1 (Appendix 1). Then, genotyping and haplotyping analysis was performed with another six microsatellite markers - D1S237, D1S419, D1S556, D1S229, D1S227, and D1S2860 - around the *USH2A* gene. The fine mapping primer sequences were obtained from the Human Genome Database (GDB). Pedigree and haplotype maps were constructed using Cyrillic V. 2.0 software.

### Mutation screening of the USH2A gene

Mutation screening was performed in all four families using direct DNA sequence analysis. The coding region (exons 2–72) and the exon-intron boundaries of *USH2A* were amplified by PCR in the probands of the four families. The pairs of primers for exons 2–72 were used according to previously published (Table 1) articles [5,9,21]. For direct sequencing, amplicons were purified (Shenneng Bocai PCR purification kit; Shenneng, Shanghai, China). An automatic fluorescence DNA sequencer (ABI, Prism 373A; Perkin Elmer, Foster City, CA), used according to the manufacturer’s instructions, sequenced the purified PCR products in both the forward and reverse directions. Nucleotide sequences were compared with the published cDNA sequence of the *USH2A* gene (GenBank NM_206933.2). For *USH2A*, cDNA numbering +1 corresponds to A in the ATG translation initiation codon in RefSeq (AY481573.1).

**Table 1:** Primer information for the USH2A gene sequencing.

**Primer**	**Forward sequence (5′-3′)**	**Reverse sequence (5′-3′)**	**Products (bp)**	**Tm (°C)**
Exon 2	GCCTGGGATGAGCTTCAG	GGTTTGGAATTCAGGCTGA	840	62
Exon 3	CACCACTGTAACTGCACAATACC	CTGCTGCAGATTTTGTGAGTAGA	345	64
Exon 4	GTCTTCCCAGCTGAACAAAGTA	GTGGTAATTTGTTCAGTAGCCCTAG	382	60
Exon 5	GTCAGGTATTGCTTGGTAAACAG	CAGCATTTATCCTTTCGGTTC	173	62
Exon 6	CGAGTGACATTCATTTGTAACGA	GGCATTTGTTGCAATAACCA	437	58
Exon 7	TTTGAATCTAATAATTCCATGGTTTG	TGGTGGTGAAGGGAAGTCTC	372	58
Exon 8	CAACATTTTGATTTCTGTTTTGC	TGCTCTGACATCTTAATGTGCT	370	62
Exon 9	CACACAATGCATATAGTCCTAGG	TGTTAGGCCAAGATTAAGTTCAT	267	62
Exon 10	TGATATGTGCTTTACTTCTGGTG	GCATTGTAGATAGAAGCACACAG	356	62
Exon 11	TGGCAGGTAGAGATGAAAGG	GCAAATGCAGTCTTCAATTCTAC	371	62
Exon 12	CCCTGTCTTGTACCTAATGAGC	TTCCAGATGGTAATAGAGATGTGA	323	62
Exon 13	GCAGTAGCATTGTTTGTGTCTC	GTAGAAGCCACAAACCAGAAAC	816	58
Exon 14	GGGAATTAGTGCCTTGGTAGAG	GAAGTTATTGCTTTGCAACTGC	379	64
Exon 15	AAGCCGTCTTACTCTACAATGCT	TTCTGATGGGTTCTAAATGGAG	360	58
Exon 16	GCAATCCTGAATCAGAAAGACC	CCACAACAGCATTTATCCTCAA	354	64
Exon 17	GAGAGGAAAGCAGTTAGCAATG	GATTCTCATTCATGTCTTGACCA	626	64
Exon 18	AAGTAACCCCTTTGTCTGATGAGT	GGAAACATTTGCATTCAGAGG	378	62
Exon 19	TCAGAAACTAAATGAATGTGTGA	TGCCCTGTTTAATCAATATAGAG	379	60
Exon 20	TGGTGGTTGGCAATAATTCC	GAGTTAGTGAGGGAGGAGAAGACA	381	64
Exon 21	AGCCATACAGATACTTGAAACC	GCTATCAAAGGGCTGAATTAG	501	62
Exon 22	CCATGCGAGTATATTGCTGTG	GCTGAGGGCAAGTCACATTAC	451	62
Exon 23	CAGGAAAGCCAGAATGATGC	CCCAAAGGCAAATTCAACAG	447	58
Exon 24	CAGGCAATGAGGAGAGAGGA	CCTAAAGGAAATTTTTGGCACA	371	58
Exon 25	TGAATCATTAAGAGGCTTGCAG	TGTGTGCACCATTGGAATAAC	459	58
Exon 26	GGTCTTTCTGTCACTTCTGTGC	TTCAGTTATCAGGCGTGGTG	722	62
Exon 27	TGCTTTCAGGGAACTGTTTTG	GGTGCTGCTTTTAGCCTGAG	497	62
Exon 28,29	TGCTGCAGAGGACAAAAATG	GCTTCAGGGTAATAGTCCTTCC	579	62
Exon 30	TGCGGCCATTAAAAGTGAAG	TGCAGGCTTCCACTACTTTAG	434	58
Exon 31	GCAGAAAGGGAGGAAAATGAC	CAAATTAGGTTGGGGTTTGC	392	64
Exon 32	TGATTTATTGGTTTGGGTCTG	GCATTCTTGATTAAATTTGCAG	357	58
Exon 33	TGAAGCATCCTATATGTTATTGC	CCTCCCCTGATTGAACTCAC	392	58
Exon 34	ATTTCCTTTTTGCCCCTCAG	AGGATGGGAAGAGAGTTTTCAG	512	56
Exon 35	TTGGGGAAGTAAAGGTAGCAC	CCACAATCTCCCCAACTAGAG	431	62
Exon 36	AAATCACATCAAGAGTGCTTGC	CCTGCTTGAAAGGCTAGCTG	352	62
Exon 37	TGTGCTTTGATCCTGCTGAC	AACCAACATCTGTGGCTAAAAG	431	60
Exon 38	AATTGGCCAGGTCAACTCAG	TGTGAGGTGGATGAAAGCAG	614	64
Exon 39	CAGGAGTTCAGGAATGAAAATG	AAGTTTCAGTGGGAAGAAATCC	526	54
Exon 40	GAGATCTCATTTGATGGCAGAA	GGCTCATTTCTTTGCTTTGG	373	58
Exon 41	TGGCCTTTACCAAGTGTTCA	AAGGCCAAAACCCAGTTTCT	890	54
Exon 42	GCAAAATTCTAGGCCTCGTG	AAAGCCTCCTTCATTTCCCTAC	492	62
Exon 43	ATGCCACAGAACAGCCTGAG	AGCCGTGACAAAGGCAATAG	469	62
Exon 44	TTTTGTAGAGGGGTGGAAGG	TGTGTACATGGGGGAGGTTC	367	58
Exon 45	CATTTCCAAAACAAAGGCTCTC	TTAGCCTCCACCCCCTTC	464	58
Exon 46	TCATCATATCCCACTGGTCAC	CCCTTCTCTCTTTTCCCTTCC	599	54
Exon 47	AGGGAAGGTGGGATTCAGAC	TGTCATGGCTGAGAGGATACC	280	59
Exon 48	CCTCACGCCATGTGTTATTC	CCTTTCTTTTCCGTGGAGTC	530	54
Exon 49	TCGAATGATCCTGGAAAATACA	TTGTTGAGAGGGAGGTGTTTG	432	56
Exon 50	ACCTGTAAGTTGCCATGTGTG	TTTGGAGGACATGACCTTTTC	678	54
Exon 51	ATTTCAGCAACTGCCTGAGC	AAAGCTTCCCTGAGAACAGC	552	62
Exon 52	TGGGAAGCTGCAAAACTG	GGCCTCAAAGTATGATGGAATG	564	54
Exon 53	TCCGTATCCCTGCATCT	GGTTAGATGCATAGGGCAAT	500	56
Exon 54	ATTGCATTTCTTCCGAACAC	TCTCTCCTTCCAGCCATAGG	428	54
Exon 55	AAAGGGAAATGCTTCTCCAAG	CCCCCTAACCACAATGACAG	396	62
Exon 56	AGCCTCTTATGAGGTTCAGACC	CAAGCCTGAAGAATGGGAAC	422	54
Exon 57	GGGGATGGTGTGACTTTTG	ATGGCCAATGAATGAGGAAG	382	56
Exon 58	GGCAAAGAGTTTGCAATTTGTC	TTTATCCAGGAGACCGACTATG	399	62
Exon 59	CAAACATTTGTTGCCCATTC	GCCAGACTGTGATTTTTCTGG	488	54
Exon 60	TGCAAAAGGACAGGTTAAAA	GATTCTGCTGTGTTGGAGCA	343	54
Exon 61	TGACACCAGGAAGAAACAGC	TTATCCCCGTGACTACATTGC	638	54
Exon 62	TTTGGCCATGAGGTTCAGAG	TGAAGGGAGTTTTCCCACAG	417	60
Exon 63-A	AGGTTAAAAAGGGGCTAAGT	GGATATCACAGGTGGAGAGAGA	600	54
Exon 63-B	ATTCAGCTGGGCATACCTGT	CCATTGTCCAGGCAGATTTT	591	59
Exon 63-C	GAATGGAGGTTGCACAGCTA	GGCTCAGGCAATAGAAAGGTC	600	58
Exon 64	AATCTCGGCTCACTGCAAG	AGTGCCTTTTCAAATTGTGC	602	62
Exon 65	TGCTTTTGGTTGCCAATTTC	ACCGTAGAGCAACTGAGAACAG	440	58
Exon 66	TGTAGGAGGTGGGATCTTGG	CTGGGGAGTGCCAGGTAG	445	60
Exon 67	GAGCAGTTTCCTGCAAATGG	TCCCCACAAGAAAATCCTTC	579	56
Exon 68	GTTTGGATTGGTTCGGTTTG	CGTAAAGCTGGGGAACAGAG	344	60
Exon 69	CGTCATAACTTGCTTTGGAATC	CAACACTTGGCACAATTTCTTC	338	60
Exon 70	ATCAAATAGCAGGGCCAGAG	CCTCTCTTGGTCCCCACAC	462	60
Exon 71	GCTGCTAAATTCTGTAGGTGACA	TAAGTGCTCAGAGGCGAGTG	499	56
Exon 72	TGAGGCTTCTGAGGCTTAG	CTGCCAAACAGAACCAAGTG	651	58

### Restriction fragment length polymorphism analysis

Variations (c.2802T>G, c.8232G>C, c.3788G>A, and c.14403C>G) found in the sequencing were confirmed with the restriction endonucleases Hinc II (TaKaRa, Dalian, China), HpyCH4V, BsaI, and SpeI (New England Biolabs, Ipswich, MA), respectively, which were used in all available family members and in the100 normal controls.

### Single strand conformation polymorphism

To validate the variations (c. 1876C>T and c.7123delG) found in the sequencing, a single strand conformation polymorphism (SSCP) analysis was performed in all available family members and in the 100 normal controls. As the PCR fragments used in SSCP analysis were between 150 and 300 bp, two pairs of specific primers were designed for detecting mutations in exon 11 and exon 38 (Table 2).

**Table 2:** Primers used in single strand conformation polymorphism (SSCP), high resolution melt curve analysis (HRM), and PAGE (PAGE) analysis in this study.

**Primer**	**Sequence (5′-3′)**
U11 SSCP	F: TGATGCAGGAAGGAACTGTG
U11 SSCP	R: CCTGGCAAATGCAGTCTTC
U32 HRM	F: ATCCCTTCCCAGTTCTTTG
U32 HRM	R: CAGATATGGAACCCCTGGAT
U38 SSCP	F: AATTGGCCAGGTCAACTCAG
U38 SSCP	R: GCACCCAAAGGTTTGTCTC
U48 PAGE	F: TGGTATCCATGCGCTAAAAC
U48 PAGE	R: CACTTGGAGTCTTGAGTGAGAA

Abbreviations: U represents USH2A; the number represents the name of the exon; F represents forward; R represents reverse.

### High-resolution melt curve analysis

To confirm the variation (c.6249delT) found in the sequencing, a high-resolution melt curve analysis (HRM) was performed in the available family members and in the 100 normal controls. Primer sequences were designed to obtain the best HRM performance, avoiding hairpin and primer–dimer formation as much as possible, and keeping the amplicon length under 250 base pairs. One pair of specific primers was designed for detecting a mutation in exon 32 (Table 2). The 10 μl reaction mixture consisted of 5 μl SsoFast EvaGreen Supermix (Bio-Rad Laboratories, Hercules, CA), 1 μl genomic DNA (10–150 ng/μl), 0.5 μl forward primer (10 pmol/μl), 0.5 μl reverse primer (10 pmol/μl), and 3 μl double distilled water. PCR cycling and an HRM analysis were performed on the Rotor-Gene 6000^TM^ (Corbett Research, Mortlake, NSW, Australia) [22].

### Bioinformatics analysis

Garnier-Osguthorpe-Robson (GOR) software was used to predict the effect of the mutation on the secondary structure of USH2A [23]. This method infers the secondary structure of a sequence by calculating the probability for each of the four structure classes (helix, sheet, turn, and loop), based on the central residue and its neighbors from the calculated matrices [23].

## Results

### Clinical findings

**Figure 1:**
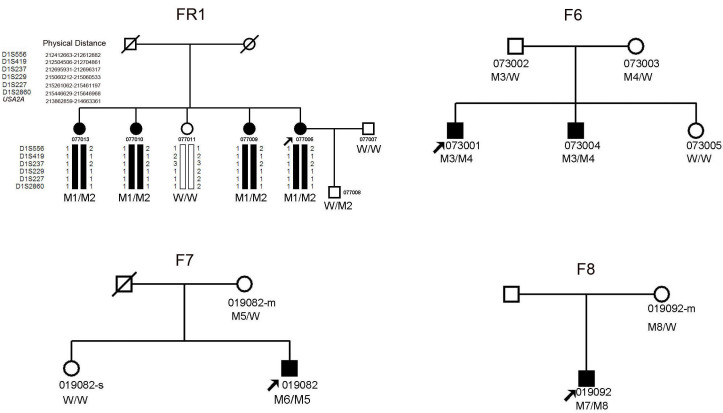
The pedigrees of the four Chinese families with autosomal recessive retinitis pigmentosa (arRP) or Usher syndrome type II (USH2), and with mutations in the *USH2A* gene. Pedigree and haplotyping analyses of family one (FR1) showed segregation with six microsatellite markers on chromosome 1 listed in descending order from the centromeric end. Squares indicate males; circles indicate females; slashed symbols indicate deceased; solid symbols indicate affected; open symbols indicate unaffected; and arrow symbol indicates proband. The genotype of each evaluated individual is shown below the individual’s symbol and identification number. Abbreviations: Wild type (W); p.C934W (M1); p. W2744C (M2); p. R626X (M3); p. I2084fs (M4); p. W1263X (M5); p. D3165fs(M6); p.G2375fs (M7); p.Y4801X (M8).

**Figure 2:**
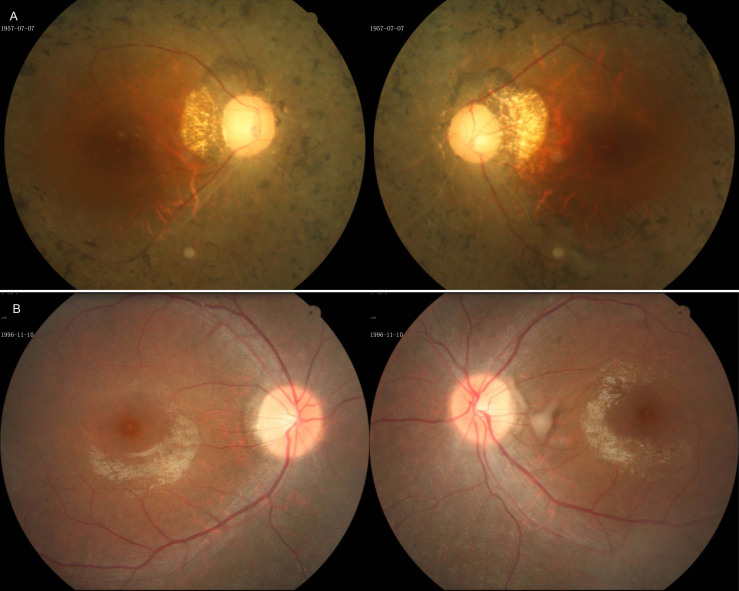
The appearance of the fundus in two patients with non-syndromic retinitis pigmentosa (RP) or Usher syndrome type II (USH2). **A**: Fundus appearance of patient 077066 from family one (FR1), shows typical retinal degeneration with attenuation of the retinal vessels, irregular pigment clumps in the retina, and waxy pallor of the optic nerve head. **B**: Fundus appearance of patient 019092 from family F8.

This study identified one Chinese family, consisting of four patients and one unaffected relative, diagnosed with non-syndromic RP, and three unrelated Chinese families, including four patients and six unaffected relatives diagnosed with USH2. The inheritance pattern in the families was autosomal recessive (Figure 1). All the patients had experienced night blindness and vision acuity impairment. The patients with USH2 had hearing impairment in early childhood. Ophthalmoscopic examination demonstrated attenuation of the retinal vessels, bone-spicule pigmentation in the fundus, and waxy pallor of the optic nerve head (Figure 2). The wave amplitudes of the ERG of the probands were indistinguishable from the baseline. Audiometric tests indicated moderate to severe sensorineural hearing impairment in the patients with USH2; in contrast, the results from the patients with non-syndromic arRP were normal. Vestibular functions of all the patients were normal. The detailed clinical information for each family’s proband is summarized in Table 3.

**Table 3:** Clinical features of the probands from the four Chinese families with retinitis pigmentosa (RP) or Usher syndrome type II (USH2)

**Family number**	**Proband**	**Best corrected visual acuity (R/L)**	**Oneset age of night blindness (year)**	**Fundus appearance**	**Oneset age of hearing loss (year)**	**Hearing impairment**	**Cataract**	**Visual field**	**ERG**	**Vestibular function**
FR1	077006	0.4/0.4	25	RP	Normal hearing	Normal	Both eyes	N/A	Wave undetectable	Normal
F6	073001	0.5/0.4	13	RP	5	Moderate(sp)	No	N/A	N/A	Normal
F7	019082	1.0/1.0	17	RP	1	Moderate(sp)	No	10°	N/A	Normal
F8	019092	0.6/0.6	12	RP	8	Moderate(sp)	No	10–15°	Wave undetectable	Normal

Abbreviations: R represents right eye; L represents left eye; SP represents slight progressive; N/A represents data not available.

### Genotyping results

Family FR1 was genotyped with 50 polymorphic markers around the known arRP loci. The mapping results excluded the other known arRP loci with the exception of the *USH2A*. Further genotyping and haplotyping analysis for the six markers (D1S237, D1S419, D1S556, D1S229, D1S227, and D1S2860) suggested that the *USH2A* gene might be the disease-causing gene in this family (Figure 1).

### Mutation analysis

**Figure 3:**
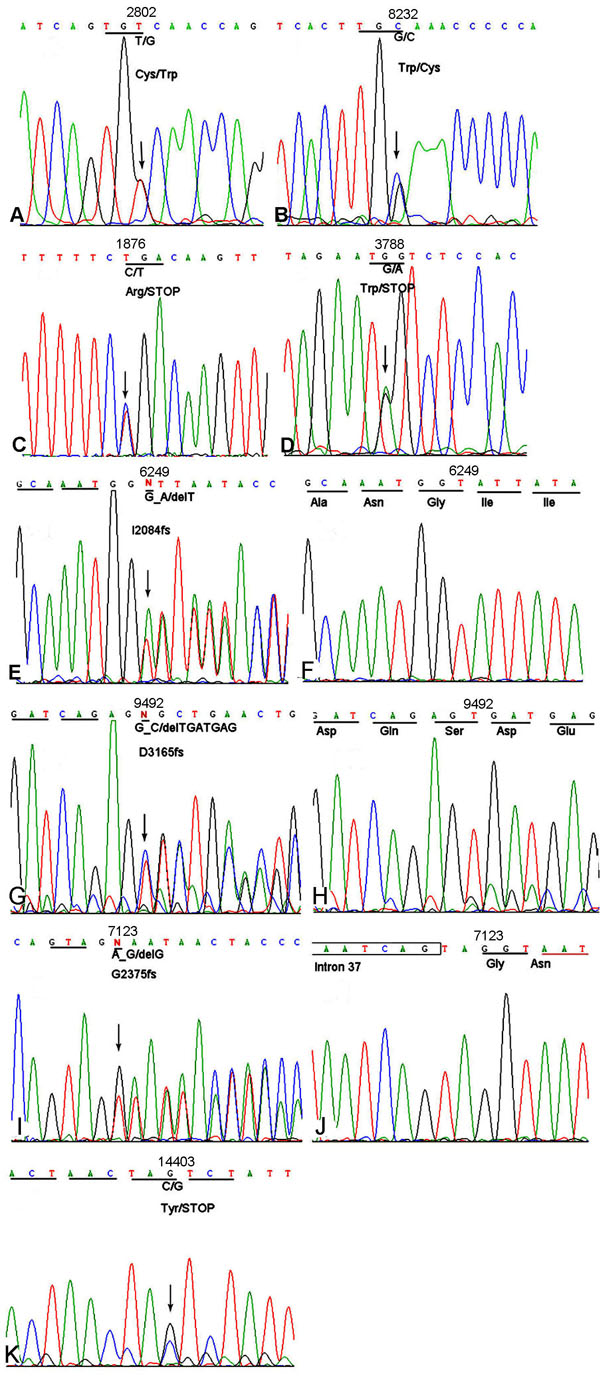
A direct sequencing analysis of the coding region of the Usher syndrome type IIA (*USH2A*) gene. **A**: Sequence presents the heterozygous missense mutation c.2802T>G (p.C934W) detected in patient 077006. **B**: Sequence shows the heterozygous missense mutation c.8232G>C (p.W2744C) identified in patient 077006. **C**: Sequence presents the heterozygous nonsense mutation c.1876C>T (p.R626X) identified in patient 073001. **D**: Sequence shows the heterozygous nonsense mutation c.3788G>A (p.W1263X) detected in patient 019082. **E** shows the heterozygous, one-base-deletion mutation c.6249delT (p. I2084fs) in patient 073001; **F** is the corresponding wild-type sequence. **G** presents a heterozygous 7 bp deletion mutation c.9492_9498del TGATGAT (p. D3165fs) in patient 019082; **H** shows the corresponding wild-type sequence. **I** presents the heterozygous, one-base-deletion mutation c.7123delG (p. G2375fs) in patient 019092; **J** shows the corresponding wild-type sequence. **K**: Sequence shows the heterozygous nonsense mutation c.14403C>G (p. Y4801X) detected in patient 019092.

**Figure 4:**
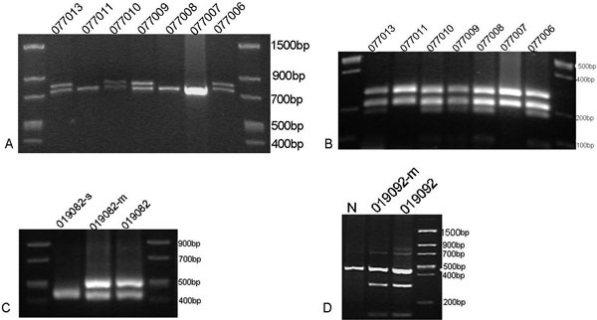
A restriction fragment length analysis of the four mutations detected in this study. **A**: c.2802T>G abolished a HincII restriction site that co-segregated with the affected individuals and the carriers (42 bp, 57 bp, 99 bp, 717 bp, and 774 bp), but not with unaffected individuals and normal controls (42 bp, 57 bp, and 717 bp). **B**: c.8232G>C created a new HpyCH4V restriction site that co-segregated with the affected individuals and the carriers (88 bp, 186 bp, 218 bp, and 274 bp), but not with unaffected individuals and normal controls (218 bp, 274 bp). **C**: c.3788G>A abolished a BsaI restriction site that co-segregated with the affected individuals and the carriers (70 bp, 132 bp, 422 bp, and 492 bp), but not with unaffected individuals and normal controls (70 bp, 132 bp, and 422 bp). **D**: c.14403C>G created a SpeI restriction site that co-segregated with the affected individuals and the carriers (145 bp, 300 bp, and 445 bp), but not with unaffected individuals and normal controls (445 bp). A participant identification number is given above each lane. N represents normal controls.

**Figure 5:**
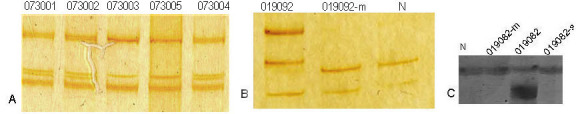
A single-strand, conformation, polymorphism analysis and a 16% denaturing polyacrylamide gel electrophorese analysis. **A**: Single strand conformation polymorphism (SSCP) analysis for the heterozygous mutation c. 1876C>T revealed that the mutant pattern (four bands) co-segregated with the affected individuals and carriers, but not with the unaffected individuals and normal controls (three bands). **B**: SSCP analysis for c.7123delG showed that the mutant pattern (three bands) co-segregated with the affected individuals and carriers, but not with the unaffected individuals and normal controls (two bands). **C**: 16% denaturing polyacrylamide gel electrophorese analysis for the heterozygous mutation c.9472_9498delTGATGAG (p. D3165fs) revealed that the mutant pattern (two bands) co-segregated with the affected individuals, but not with the unaffected individuals and normal controls (one band). Participant identification numbers are listed above each lane and N represents the normal controls.

**Figure 6:**
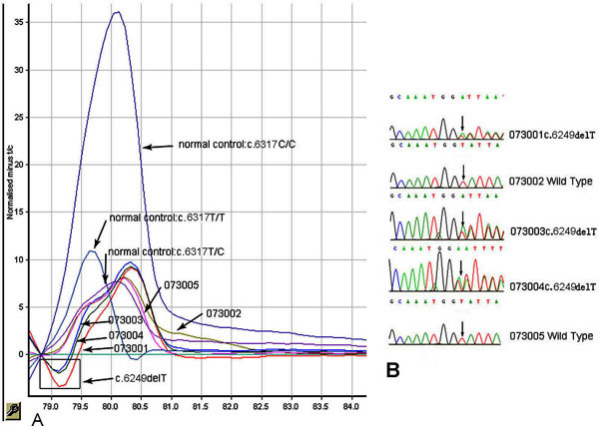
A high-resolution, melt curve analysis (HRM) for the mutation c.6249delT (p. I2084fs) in family F6. In the amplicon, there is a SNP rs6657250, c.6317T>C, as marked in the plot. **A**: A difference plot for the five members in family F6. The median green, straight line presents the normal control with c.6317T>C. The real-time PCR products of the family members are compared to the median normal control to produce the plot. The curve revealed that the mutant pattern (area within the rectangle) co-segregated with the affected individuals 073001, 073004, and carriers, 073003, but not with the unaffected individuals 073002, 073005, and normal controls c.6317T/T or c.6317C/C. **B**: Direct sequencing analysis shows the heterozygous, one-base-deletion mutation c.6249delT (p.I2084fs) in patients 073001 and 073004 and in the carrier 073003; sequences in individuals 073002 and 073005 are the wild type.

Sequencing of the *USH2A* gene revealed 17 sequence variants in this study, eight of which were pathogenic mutations (Table 4). All eight pathogenic mutations were heterozygous; seven of them were first detected in the current study (Figure 3 and Table 4). Using RFLP, SSCP, or HRM analysis, the eight mutations co-segregated with the RP/USH2 phenotype, respectively (Figure 4, Figure 5, Figure 6). Analyses did not detect the other seven mutations in the 100 normal controls, with the exception of p.C934W, which was identified in its heterozygous state in two individuals among the 100 normal controls (Table 4).

**Table 4:** Disease-causing mutations in the Usher syndrome type IIA (USH2A) gene identified in this study

**DNA change**	**Exon**	**Protein change**	**Type of nucleotide change**	**Family number**	**Frequency**	**Source**
c.2802T>G	13	p.C934W	Heterozygous	FR1	2/200	This study
c.8232G>C	42	p.W2744C	Heterozygous		0/200	This study
c.1876C>T	11	p.R626X	Heterozygous	F6	0/190	[24]
c.6249delT	32	p.I2084fs	Heterozygous		0/200	This study
c.3788G>A	17	p.W1263X	Heterozygous	F7	0/200	This study
c.9492_9498delTGATGAT	48	p.D3165fs	Heterozygous		0/200	This study
c.7123delG	38	p.G2375fs	Heterozygous	F8	0/200	This study
c.14403C>G	66	p.Y4801X	Heterozygous		0/200	This study

The “Frequency” column, shows the number of chromosomes.

**Figure 7:**
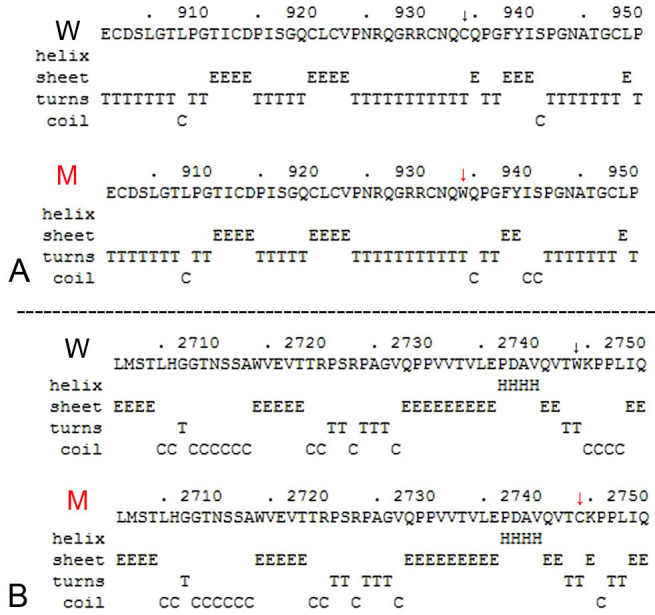
The effect of p.C934W and p. W2744C on the secondary structure of the USH2A using the GOR method. W denotes wild type, M denotes mutant.

Four different combinations of heterozygous mutations were detected in the four families. In family FR1 (non-syndromic arRP), two missense mutations, c.2802T>G (p.C934W) and c.8232G>C (p.W2744C), were detected in different alleles of patient 077006 (Figure 1, Figure 3, Figure 4). Using the GOR method, the results for secondary structure prediction suggested that p.C934W replaced two β sheets “E” with two coils “C” at amino acids 935 and 940, respectively. Mutation p.W2744C substituted a β sheet “E” and two turn sheets “T” for three coils “C” at amino acid 2745, 2747, and 2748, respectively (Figure 7). For the three USH2 families (F6, F7, and F8), one allele carried nonsense mutations, c.1876C>T (p.R626X), c.3788G>A (p.W1263X), and c.14403C>G (p. Y4801X), respectively, while the other allele harbored deletion mutations c.6249delT (p. I2084fs), c.9492_9498del TGATGAT (p. D3165fs), and c.7123delG (p. G2375fs), respectively (Figure 1, Figure 3, Figure 4, Figure 5, Figure 6).

In addition to the eight pathogenic mutations detected in this study, nine nonpathogenic sequence variants were also identified. Table 5 summarizes these variants based on their nature and frequency.

**Table 5:** Presumed nonpathogenic variants of the Usher syndrome type IIA (USH2A) gene found in this study.

**Exon**	**Nucleotide change**	**Codon**	**rs number**	**Family number**	**Allele frequency**	**Source**
2	c.373A>G	p.A125T	rs10779261	F6	N/A	[14]
3	c.504A>G	p.T168T	rs4253963	F7	267/720	[20]
21	c.4457A>G	p.K1486R	rs1805049	F7	76/180	[24]
28	IVS27–34delC		rs71556647	FR1	N/A	[c]
32	c.6317T>C	p.I2106T	rs6657250	FR1,F6,F7,F8	N/A	[29]
34	c.6506T>C	p.I2169T	rs10864219	FR1,F8	27/100	[15]
48	IVS48+78C>T			FR1	N/A	This study
52	c.10232A>C	p.E3411A	rs10864198	FR1	23/64*	[27]
63	c.12612G>A	p.T4204T	rs2797235	FR1,F8	N/A	[27]

Abbreviations: N/A represents data not available; the asterisk indicates that the allele frequency referred to patients.

## Discussion

This study detected eight different mutations of the *USH2A* gene isoform b in one non-syndromic arRP family and in three USH2 families. Scandinavian, French, European, and Canadian studies [12,14,16,24-26] previously reported the nonsense mutation p.R626X. The remaining seven mutations were first identified in this study.

**Figure 8:**
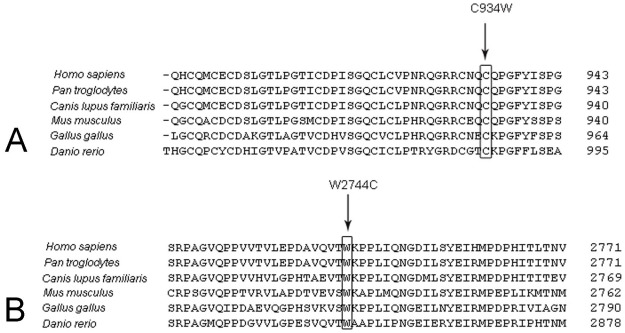
The sequence alignment portion of **A**: Laminin epidermal growth factor (EGF)-like domain and **B**: Fibronectin type 3 domain, in the long usherin isoform from different species. Both Cys934 (C934) and Trp2744 (W2744) are conserved during evolution. The arrows indicate the mutated residues in Usher syndrome type IIA (USH2A).

Rivolta et al. first reported that about 4.5% of 225 patients from North America with non-syndromic recessive RP carried the missense mutation p.C759F [12]. Then, Bernal et al. found that there was a similar detecting frequency (4.6%) for p.C759F in Spanish patients [13].Two novel missense mutations, p.C934W and p.W2744C, were found in family FR1. Although p.C934W was identified (in a heterozygous state) in two individuals among the 100 normal controls, both mutations have been classified as deleterious-effect missense mutations with several lines of evidence. Both mutations co-segregated with the phenotype of family FR1 and both residues (C934 and W2744), located in the 8th Lam EGF domains and in the 14th FN3 repeat of the usherin, respectively, were highly conserved in different species (Figure 8). The results of GOR suggested that p.C934W and p.W2744C lead to secondary structure changes around residues 934 and 2744, which might interfere with the correct folding of the usherin (Figure 7). As the results of audiometric tests for the patients from family FR1 were normal, the two compound missense mutations might be responsible for RP without hearing loss.

Three different compound heterozygous mutations were identified in three families (F6, F7, and F8) with USH2 and all six mutations directly or indirectly resulted in premature termination of the USH2A translation. This is consistent with Dreyer et al. [25] previous observation that patients carrying compound heterozygous mutations (either two truncating or one truncating combined with one missense) in exon 22–72 presented the Usher type II phenotype. In contrast to the patients from the three USH2 families, the patients in FR1 carried two missense mutations. A recent study in a cohort of 272 Spanish patients with non-syndromic RP resulted in the identification of two mutant alleles of the *USH2A* gene in nine patients, with seven of them carrying either homozygous missense mutations or two heterozygous missense mutations [18]. In a large Chinese family, four patients carrying one truncating combined with one missense mutation (p.G1734R) exhibited RP with hearing loss, while the only person harboring the homozygous misense mutation (p.G1734R) presented RP without hearing loss [19]. However, this phenomenon was not observed in one Israeli family with three non-syndromic RP patients carrying one missense mutation and one truncating mutation [15].

**Figure 9:**
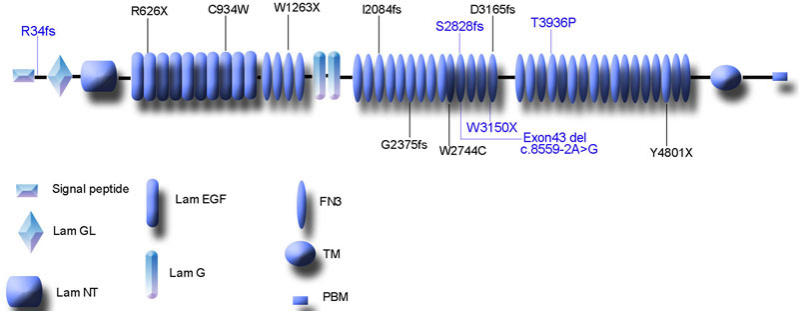
A schematic distribution showing the eight disease-causing mutations in the Usher syndrome type IIA (USH2A) isoform b protein domains identified in this study and in the five disease-causing mutations (blue characters) from a previous study [21]. Abbreviation: Laminin G-like domain (Lam GL); Laminin N-terminal (Lam NT); Laminin EGF-like domain (Lam EGF); Fibronectin type III (FN3); Laminin G domains (Lam G); Transmembrane region (TM); and PDZ-binding motif (PBM).

As in our previous study [21], with the exception of one mutation (p.R626X), the other mutations identified in the current study were novel and were spread relatively evenly along the *USH2A* gene (Figure 9). These results indicate that the mutation spectrum for the *USH2A* gene among Chinese or Asian patients differs from the mutation spectrum among European Caucasians. The common mutations, p. E767fs for USH2 and p.C759F for arRP in Caucasians, are not detected in Chinese and Japanese patients [12-14,16,18,19,21,27-29].

In conclusion, our results further support previous indications that the mutations of the *USH2A* gene are also responsible for non-syndromic RP in Chinese patients. The mutation spectrum among Chinese patients appears to differ from that among European Caucasians.

## Appendix 1. 50 markers used in the known arRP genotyping.

To access the table, click or select the words “Appendix 1.” This will initiate the download of a pdf archive that contains the table.
